# Efficacy of Preoperative Music Intervention on Pain and Anxiety in Patients Undergoing Cataract Surgery

**DOI:** 10.3389/fphar.2021.748296

**Published:** 2021-09-30

**Authors:** Gilles Guerrier, Federico Bernabei, Mathieu Lehmann, Marco Pellegrini, Giuseppe Giannaccare, Pierre-Raphaël Rothschild

**Affiliations:** ^1^ Anaesthetic and Intensive Care Department, Hôpital Cochin, Paris Descartes University, Paris, France; ^2^ Department of Ophthalmology, Hôpital Cochin, Assistance Publique-Hôpitaux de Paris, Paris, France; ^3^ Ophthalmology Unit, DIMES, University of Bologna, IRCCS Azienda Ospedaliero Universitaria di Bologna, Bologna, Italy; ^4^ Department of Ophthalmology, University Magna Graecia of Catanzaro, Catanzaro, Italy; ^5^ Université de Paris, Centre de Recherche des Cordeliers, INSERM, Paris, France

**Keywords:** cataract surgery, pain, music intervention, music therapy, anxiety

## Abstract

The aim of the present study was to investigate the impact of preoperative music exposure on intra- and post-operative pain during cataract surgery. This study was conducted alongside a prospective single-masked randomized controlled trial (ClinicalTrials.gov NCT02892825). Patients undergoing first eye cataract surgery were included and randomly assigned to either the intervention or control group. Patients in the intervention group had a 20-min music session through earphones before surgery, while patients in the control group wore earphones without music. Anxiety level evaluated using the visual analog scale and heart rate were collected before and after music intervention. Pain level was assessed using the Numerical Pain Rating Scale, during the surgical procedure, prior to discharge and 7 days postoperatively. A total of 243 patients were included: 119 in the intervention group and 124 in the control group. No significant differences in baseline characteristics, including age, sex and rate of treated hypertension were found between the 2 groups (all *p-values* > 0.05). In addition, no significantly differences were found in heart rate and anxiety level before music intervention between the 2 groups (all *p-values* > 0.05). Conversely, anxiety level was significantly lower in the music group after the intervention (respectively, 1.3 ± 1.1 vs 3.2 ± 2.2; *p* < 0.05). Patients in the music group reported a lower mean pain level during surgical procedure and before discharge compared with control group (respectively, 1.2 ± 0.5 vs 2.1 ± 1.1, *p* = 0.03 and 0.23 ± 0.4 vs 0.81 ± 0.7, *p* = 0.04). No difference was found in pain level 7 days postoperatively (0.1 ± 0.3 vs 0.2 ± 0.4, *p* = 0.1). A significant correlation was found between anxiety level and intraoperative pain level (*R* = 0.64, *p* = 0.02). In conclusion, music intervention was effective in reducing anxiety level and self-reported pain both during surgery and in the early postoperative period.

**Clinical Trial Registration:** https://clinicaltrials.gov/ct2/home, identifier NCT02892825.

## Introduction

Cataract surgery represents one of the most commonly performed procedures in the world (Pascolini and Mariotti, 2012). Advancements in anesthesia and surgical techniques made it possible to perform most of the procedures for cataract extraction under topical anesthesia (Pellegrini et al., 2020; Lundström et al., 2021). On the one hand, this achievement has greatly reduced operating times and side effects related with local or general anesthesia (Giannaccare et al., 2021; Lundström et al., 2021). On the other hand, despite sedation (e.g. benzodiazepines and opioids) which could be administered pre- and intra-operatively, patients may experience a state of considerable anxiety along with a certain level of pain and discomfort during the surgical procedure (Rothschild et al., 2013; Shi et al., 2019).

Preoperative music intervention is a non-expensive and easily applicable technique with no side effects that showed significant beneficial effects on patients’ anxiety in different surgical populations (Choi et al., 2018; Wan et al., 2020; Kakar et al., 2021). In addition, music intervention has been proved to lead to a better patient’s cooperation and a reduced intraoperative blood pressure during surgery under topical anesthesia (Choi et al., 2018; Fu et al., 2020; Muddana et al., 2020).

Although the underlying mechanism of music therapy remains still unclear, it has been demonstrated that it induces molecular changes related to opiates and cytokine processes (Stefano et al., 2004). Different psychophysiological mechanisms have been proposed, and in particular music would be effective in distracting patients from the surgical procedure, mainly when they listen to music of their preference (Gaberson, 1995; Allen et al., 2001; Clements-Cortes and Bartel, 2018).

Previous studies demonstrated the beneficial effect of music intervention on both anxiety and blood pressure in patients undergoing cataract surgery (Leo et al., 2003; Wiwatwongwana et al., 2016; Muddana et al., 2020). However, there is little knowledge regarding the effect of preoperative music therapy on intra- and post-operative subjective pain in this patient population (Choi et al., 2018).

Interestingly, it has been demonstrated that pre-operative anxiety is positively correlated with intra- and post-operative pain level in a variety of surgical populations (Robleda et al., 2014; Bandeira et al., 2017). Therefore, it is possible to speculate that music intervention may result in beneficial effect on pain level in patients undergoing cataract surgery. Thus, the aim of the present study was to investigate the effect of music intervention on self-reported pain intensity during first eye cataract surgery and in the early post-operative period.

## Materials and Methods

### Design and Patients

This study was conducted between February 2017 and July 2018 at the Ophthalmology service, OphtalmoPôle de Paris of the Cochin Hospital (Paris, France), alongside a prospective single-masked randomized controlled trial aiming at evaluating the effect of music intervention on anxiety-induced hypertension during cataract surgery performed under local anesthesia (ClinicalTrials.govIdentifier: NCT02892825). The study was performed in accordance with the principles of the Declaration of Helsinki and was approved by the Comité de Protection des Personnes Paris-Ile-de-France III (N°2016-A00728-43). Written informed consent was obtained from all study subjects. Patients scheduled for first eye cataract surgery under local anesthesia were screened to be enrolled in the study. Exclusion criteria were hearing loss, speech impairment, uncontrolled hypertension, psychiatric disorders, dementia, deprivation of liberty by judicial or administrative decision or under legal protection. Uncontrolled hypertension was defined as a systolic blood pressure >140 mmHg and/or diastolic blood pressure >90 mmHg, despite anti-hypertensive therapy, during the pre-anesthesia examination. In addition, patients with complicated cataract and hard nuclear cataracts with nuclear opalescence scores 5 or greater on Lens Opacities Classification System-III system were excluded from the study (Chylack et al., 1993).

### Music Intervention and Surgical Procedure

Patients were randomized using a computer-generated, interactive web-response system (Cleanweb^®^, Telemedecine technologies S.A.S, Boulogne-Billancourt, France) and assigned to the music or the control group. After the randomization, all patients had dedicated headphones (BOSE AE2^®^) positioned. Patients in the music group were shown how to handle a tablet interface by a trained nurse in order to choose a music program according to their preferences (MUSIC CARE^®^ Paris, France). In the control group, headphones were placed on patient’s ears, but no music was played. A sleeping mask concealing patient’s eyes was applied to all participants. In both groups, headphones and masks were left in place for 20 min and the headphones were removed before the surgical procedure. Before surgical procedure, 0.03 mg/kg oral midazolam was administered to all patients.

Cataract surgery was performed at one eye of each patient under topical anesthesia by an experienced surgeon (ML, PRR, DM). Topical anesthesia consisted in administration of oxybuprocaine 0.5% drops into the conjunctival sac 3 times in the 15 min preceding surgery. The primary steps of the surgery were a self-sealing temporal limbal 2.2 mm incision, subsequently capsulorhexis, hydrodissection and phacoemulsification in the capsular bag were performed. Finally, a foldable intraocular lens was inserted in the capsular bag. Intracameral 1 mg cefuroxime injection was administered at the end of surgery. Postoperative topical therapy consisted of fluoroquinolone eye drops for 1 week and dexamethasone eye drops for 1 month.

### Data Collection

Data regarding age, sex, ocular and medical history including presence of treated hypertension, and duration of surgical procedure were collected.

Anxiety level was measured by the anesthetist using a visual analogue scale for anxiety (VAS-A) before and after music intervention (Facco et al., 2013). The VAS-A scale is comprised of a horizontal line 100 mm long with the indication “no anxiety” to the left and “worst possible anxiety” to the right. In addition heart rate were measured using pulse oximetry (Onyx II 9550, NONIN, Plymouth, MI, United States) before and after music intervention.

Pain level was evaluated by the anesthetist using a verbally administered 0-to-10 numerical pain rating scale (NPRS), where 0 indicates “No pain” and 10 “The worst possible pain”: 1) intraoperatively, before the insertion of the foldable intraocular lens; 2) postoperatively, before the discharge, and 3) 7 days after the surgery (Hjermstad et al., 2011).

### Statistical Analysis

The SAS 9.4 statistical software (Copyright^©^ 2016 by SAS Institute Inc., Cary, NC, United States) was used for data analysis. Continuous data were presented as mean ± standard deviation, while categorical data were represented by number and percentage.

Unless otherwise specified, categorical variables were compared by a Chi-square test or Fisher’s exact test as appropriate, and continuous variables were compared by a Student’s t test or Wilcoxon-Mann-Whitney test as appropriate. In addition, the correlation between preoperative anxiety level after music intervention and intraoperative pain level was evaluated with Pearson correlation test. A *p* value <0.05 was considered statistically significant.

## Results

Two hundred forty-three patients were included in the study and were randomized to receive music intervention (intervention group: *n* = 119) or headphone with no music (control group: *n* = 124) before cataract surgery. Demographic and baseline characteristics of patients are reported in Table 1. There were no statistically significant differences in age, sex distribution, and proportion of patients with treated hypertension between the 2 groups (all *p* > 0.05).

**TABLE 1:** Baseline characteristics of patients undergoing cataract surgery.

Characteristics	Intervention group (*n* = 119) mean ± SD or n (%)	Control group (*n* = 124) mean ± SD or n (%)	*p-*value
Age (yr)	67.3 ± 10.4	68.5 ± 11.2	0.4
Sex			0.9
Female	63 (52.9)	65 (52.4)	
Male	56 (47.1)	59 (47.6)	
Treated hypertension	11 (9.2)	13 (10.5)	0.8

SD, Standard deviation.

All patients underwent uneventful cataract surgery and no significant complications were registered in the post-operative period. No difference was found in the duration of procedure between music and control group (respectively, 16.1 ± 6.5 vs 16.5 ± 6.2, *p* = 0.08).

Table 2 shows anxiety level and heart rate before and after music intervention in both groups. No significant differences were observed in heart rate before and after music intervention between the music and control group (respectively, 75.3 ± 7.6 vs 77.1 ± 9.4 bpm/min, and 67.3 ± 7.6 vs 68.7 ± 13.1 bpm/min, always *p* > 0.05). In addition, no difference was found in anxiety level between the 2 groups before music intervention (respectively, 3.2 ± 2.2 vs 3.3 ± 2.3, *p* = 0.8). Conversely, anxiety level was significantly lower in the music group compared to control group after music intervention (respectively, 1.3 ± 1.1 vs 3.2 ± 2.2, *p* < 0.001).

**TABLE 2:** Heart rate and anxiety level in the music intervention and in the control group.

Measures	Intervention group (*n* = 119) mean ± SD	Control group (*n* = 124) mean ± SD	*p*-value
Heart rate before intervention (bpm/min)	75.3 ± 7.6	77.1 ± 9.4	0.1
Heart rate after intervention (bpm/min)	67.3 ± 7.6	68.7 ± 13.1	0.3
Anxiety level before intervention (VAS)	3.2 ± 2.2	3.3 ± 2.3	0.8
Anxiety level after intervention (VAS)	1.3 ± 1.1	3.2 ± 2.2	**<0.001**

SD, Standard deviation; VAS, Visual analogue scale.

Table 3 shows intra- and postoperative mean pain level as well as its classification according to the NPRS values. In particular, pain was stratified in the following categories: 1) NPRS = 0, 2) NPRS 1-5 and 3) NPRS >5, in the 2 groups in the different timepoints (intraoperatively, postoperatively before discharge and 1 week after surgery). Patients in the music group reported a lower mean intra- and postoperative (before discharge) pain level compared with control group (respectively, 1.2 ± 0.5 vs 2.1 ± 1.1, *p* = 0.03 and 0.23 ± 0.4 vs 0.81 ± 0.7, *p* = 0.04). Conversely, no difference was found in pain level 1 week postoperatively (0.1 ± 0.3 vs 0.2 ± 0.4, *p* = 0.1).

**TABLE 3:** Pain score evaluated before and following cataract surgery.

	Intervention group (*n* = 119)	Control group (*n* = 124)	*p-*value
Pain score			
Intra-operative			
Mean ± SD	1.2 ± 0.5	2.1 ± 1.1	**0.03**
NPRS 0 [n (%)]	78 (66%)	50 (40%)	
NPRS 1–5 [n (%)]	30 (25%)	54 (44%)	
NPRS >5 [n (%)]	11 (9%)	20 (16%)	
Post-operative before discharge			
Mean ± SD	0.23 ± 0.4	0.81 ± 0.7	**0.04**
NPRS 0 [n (%)]	102 (86%)	79 (64%)	
NPRS 1–5 [n (%)]	17 (14%)	39 (31%)	
NPRS >5 [n (%)]	0	6 (5%)	
One week after surgery			
Mean ± SD	0.1 ± 0.3	0.2 ± 0.4	0.1
NPRS 0 [n (%)]	108 (91%)	107 (86%)	
NPRS 1–5 [n (%)]	11 (9%)	16 (13%)	
NPRS >5 [n (%)]	0	1 (1%)	

NPRS, numerical pain rating scale; SD, Standard deviation; n, number of patients.

A significant correlation was found between preoperative anxiety level after music intervention and intraoperative pain level (*R* = 0.64, *p* = 0.02).

## Discussion

The aim of this randomized control trial was to investigate the effect of preoperative music intervention on the level of pain and anxiety in patients undergoing first eye cataract surgery. Interestingly, patients in the music group presented a lower level of both anxiety and pain compared with controls. In particular, patients who received music intervention experienced a lower level of anxiety when they entered the operating room and, subsequently, presented a lower level of pain, both during surgery and in the immediate postoperative period.

Previous studies have investigated the effect of pre and peri-operative music intervention on anxiety in patients undergoing ophthalmic surgery, and in particular cataract surgery (Cruise et al., 1997; Allen et al., 2001; Wiwatwongwana et al., 2016; Muddana et al., 2020). Cruise and co-authors evaluated the effect of relaxing music in patients undergoing cataract surgery under peribulbar anesthesia, demonstrating an increased satisfaction in patients who received music intervention (Cruise et al., 1997). More recently, 2 studies evaluated the effect of music intervention administrated both, before and during cataract surgery under topical anesthesia (Wiwatwongwana et al., 2016; Muddana et al., 2020). Both showed that music exposure significantly reduces anxiety and blood pressure, resulting in a better patient’s experience. The beneficial effect of music on the preoperative anxiety has also been demonstrated for other types of surgery. Interestingly, music has a long-lasting effect and in particular, it was showed that 15 min of music intervention before the surgical procedure are able to lead to an effective reduction of anxiety (McClurkin and Smith, 2016).

Several reports showed that preoperative anxiety is positively correlated with postoperative pain, in different surgical populations (Bayrak et al., 2019; Navarro-Gastón and Munuera-Martínez, 2020). Indeed, it has been shown that the analgesic effect due to the musical intervention can be helpful in reducing different types of pain, particularly in patients who have to undergo orthopedic, urological and general surgery procedures, and in patients who suffer from chronic pain (Hyung, 2016).

In agreement with this data, we found that intraoperative pain was positively correlated with preoperative anxiety level, and both were reduced in the subjects in the music group. Previously, only one study evaluated the effect of music on pain in this peculiar patient population (Choi et al., 2018). In particular, Choi and co-authors evaluated the effect of Korean traditional music before and during cataract surgery, demonstrating its effectiveness in reducing painful perception (Choi et al., 2018). Our study supports these results and furthermore, we showed that this also applies when the patient chooses the music he/she prefers. Interestingly, a recent study showed that the reduction in pain associated with music exposure is greatest when the subject chooses the music to listen to (Howlin and Rooney, 2021). The study also shows that it is the act of making a choice that determines the greatest effectiveness of the procedure, empathizing the importance of giving patients as much control as possible in music intervention (Howlin and Rooney, 2021).

Unlike previous studies, in the present one, patients were exposed to music only before surgery. This is because, in our opinion, be exposed to music during the procedure could prevent the surgeon from communicating with the patient and vice versa, thus causing a reduction in patient compliance and maybe an increase in anxiety. Cataract surgeons are aware that due to the pain related with the surgical procedure under topical anesthesia, patients can become uncooperative and make abrupt movements that can potentially cause intraoperative complications (Zhu et al., 2021). However, the use of topical anesthesia averts many of the potential systemic and ocular complications associated with regional anesthesia (Maharjan et al., 2021). For these reasons, intraoperative pain control plays a key role for the success of the surgical act. Music intervention is a safe, inexpensive, and easy-to-use procedure that should be strongly encouraged in order to improve the experience and satisfaction of the patient undergoing cataract surgery. We identified some limitations for the present study that deserve mentioning. Firstly, although they are recognized as valid indicators and are widely used in clinical trials, anxiety and pain rating scales remain highly subjective. Secondly, the surgeon’s experience was not evaluated, and it must be acknowledged that this information could have helped to better understand the effect of music during surgery. Thirdly, the cataract grading and ultrasound energy consumption during phacoemulsification were not evaluated in the present study. Although the duration of cataract surgery did not change significantly between the two groups, the evaluation of these two parameters would provide useful information to more accurately assess the effectiveness of music intervention. Further studies evaluating these parameters are warranted to better address this issue. Finally, the use of midazolam may have partially hampered the results. Although the use of the same dose of the drug in all included patients limited the possibility of biasing the results, future sedative-free studies are needed to confirm our findings.

In conclusion, the present study demonstrates that 20 min of music intervention before surgery are effective in reducing anxiety and pain sensation in patients undergoing cataract surgery. Further studies are needed to establish the best approach, in terms of timing, cost and technology, before this intervention can be widely introduced in routine cataract surgery.

## Data Availability

The original contributions presented in the study are included in the article/supplementary materials, further inquiries can be directed to the corresponding author.
